# Evaluation of an Infant Formula with Large, Milk-Phospholipid Coated Lipid Droplets on Long-Term Growth and Development of Singaporean Infants: Randomized Controlled Trial Protocol

**DOI:** 10.3390/nu13082865

**Published:** 2021-08-20

**Authors:** Lynette P. Shek, Yap Seng Chong, Antoinette Winokan, Marieke Abrahamse-Berkeveld, Eline M. Van Der Beek, Oon Hoe Teoh, on behalf of the VENUS Working Group

**Affiliations:** 1 Singapore Institute for Clinical Sciences, Agency for Science, Technology and Research, Singapore 119228, Singapore; paeshekl@nus.edu.sg (L.P.S.); obgcys@nus.edu.sg (Y.S.C.); 2 Department of Paediatrics, Yoo Loo Lin School of Medicine, The National University of Singapore and National University Health System, Singapore 119228, Singapore; 3 Department of Obstetrics and Gynaecology, Yoo Loo Lin School of Medicine, The National University of Singapore and National University Health System, Singapore 119228, Singapore; 4 Nutricia Research, Singapore 138671, Singapore; antoinette.winokan@danone.com; 5 Danone Nutricia Research, 3584 CT Utrecht, The Netherlands; 6 Department of Paediatrics, University Medical Center Groningen, University of Groningen, 9700 RB Groningen, The Netherlands; e.m.van.der.beek@umcg.nl; 7 Department of Paediatrics, KK Women’s and Children’s Hospital, Singapore 229899, Singapore; teoh.oon.hoe@singhealth.com.sg

**Keywords:** lipid droplet structure, infant growth, safety, body composition development, cohort-based recruitment, randomized controlled trial

## Abstract

A concept infant formula (IF) was developed with physical properties of lipid droplets mimicking more closely those in human milk. This paper describes the unique design of a randomised controlled trial evaluating the impact of the concept IF on infant growth and body composition development whilst applying a cohort-like recruitment approach that fully supports breastfeeding practices of the study population. Subjects entered the study between birth and 1 months of age, and whenever parents decided to introduce formula were randomised to one of three study formulas; the concept IF comprising large lipid droplets coated by milk phospholipids and containing a specific mixture of prebiotics, a standard IF with the specific prebiotic mixture or a standard IF without the prebiotic mixture. The primary objective was to evaluate the impact of the concept IF on growth and body composition outcomes during the first year of life with a follow-up at 2, 3, 4 and 5 years of age. In addition, stool, saliva and buccal smear samples and parameters assessing safety, gastrointestinal tolerance and cognitive outcomes were collected. The applied cohort-like enrolment approach is distinctly different from standard clinical safety or efficacy studies and may provide valuable insights on trial design for the evaluation of IF while carefully considering breastfeeding practices.

## 1. Introduction

Breastfeeding is the best source of nutrition to support infant growth and development [1,2]. 

Breastfeeding confers health benefits for mother and infant and, as such, has been associated with a reduced risk of obesity and cardiometabolic diseases in later life compared to infant formula feeding [3,4,5,6], potentially driven by differences in early growth and developmental trajectories [7,8,9]. Lipids are crucial in fulfilling nutritional needs: the lipid fraction of milk provides almost half of the caloric intake that infants need [10,11]. Human milk (HM) contains large lipid globules (mode diameter 4–10 μm) with a complex structure of a multi-layer membrane containing functional lipids and proteins surrounding a triglyceride core [12]. In contrast, standard infant formula (IF) contains small lipid droplets (mode diameter < 0.5 μm) and mostly proteins at the lipid-water interface without any membrane [13]. The specific surface composition and size of the lipid globules in HM was reported to play a role in gut maturation, as well as the development of the immune and central nervous systems [12,14,15,16]. To mimic the physical properties of HM more closely, a concept IF was developed containing large milk phospholipid-coated lipid droplets (mode diameter 3–5 µm; Nuturis^®^) [12]. Preclinical nutritional programming studies demonstrated that exposure to a concept IF based diet in mice from day 15 to 42 reduced body fat accumulation during adulthood by preventing adipocyte hypertrophia and improved metabolic profile with a reduction of fasting plasma leptin, resistin, glucose and lipids as well as specific cognitive behaviours [17,18,19]. It was shown that both the size and the milk-phospholipid coating of the lipid droplets in the concept IF, but not the mere presence of milk phospholipids, contributed to its reported protective effect against obesity in later life [20]. In a proof-of-concept study in adult men aimed to evaluate metabolic effects of the concept IF, earlier peak concentrations of glucose and insulin were observed after consuming the concept IF, and postprandial triacylglycerol concentrations tended to increase faster compared to consuming a standard IF [21]. Hence, together with the aforementioned preclinical studies, it was postulated that introducing large, phospholipid-coated lipid droplets might bring the physiologic properties of infant formula closer to those of human milk, potentially with lasting beneficial impact on infant growth and (metabolic) health outcomes in later life. 

Recently, we showed that the concept IF containing large, milk phospholipid–coated lipid droplets comprising a mix of dairy and vegetable lipids was safe, well-tolerated and supported an adequate growth in healthy infants up to 4 months of age in a randomised clinical equivalence study [22]. The World Health Organization (WHO) recommends exclusive breastfeeding for infants up to 6 months and continued breastfeeding thereafter, preferably until 2 years of age [23]. We designed a randomised, controlled intervention trial with careful consideration of the UNICEF Baby Friendly Initiative, aiming to fully support breastfeeding practices [24]. To this end, subjects could enter the study within the first month of life regardless of if and when the parents intended to introduce formula into their infant diet. After enrolment in the study, parents were encouraged to continue breastfeeding; infants were only to be randomised to one of the intervention formulas whenever parents decided to introduce formula at any time within the first year of life. Using this unique study design, we anticipated having a better reflection of real-life formula use, allowing for variations in timing and extent of exposure, e.g., start and duration of formula feeding during the first year of life, compared to conventional designs for clinical evaluation of infant formulas.

The primary aim of the current study was to evaluate the safety and efficacy as well as the long-term impact of the concept IF with large, milk phospholipid-coated lipid droplets containing a lipid blend of only vegetal origin compared to a standard IF on infant growth and body composition development in healthy term infants in Singapore using a cohort-like recruitment approach. It was hypothesized that the concept formula is safe and may support growth trajectories closer to those of breastfed infants compared to the standard formula. In addition, as an explorative aim, we evaluated the specific prebiotic mixture of short-chain galacto-oligosaccharides and long-chain fructo-oligosaccharides (scGOS/lcFOS; 9:1), present in both standard and concept IF and previously shown to have beneficial effects on stool characteristics, gut microbiota and immune function [25,26,27,28]. 

## 2. Materials and Methods

### 2.1. Study Objectives

The primary aim of the study was to evaluate the safety and efficacy of the concept IF with large, milk phospholipid-coated lipid droplets in healthy term infants in Singapore. In line with the aforementioned aims of the study, 4 objectives were defined: (i) examine safety by evaluation of growth equivalence during the first 4 months of age (daily weight gain as primary outcome) as well as tolerance and adverse event outcomes up to 12 months between infants fed the concept formula or standard formula, (ii) examine differences in growth outcomes between birth and 12 months (weight gain 0–12 months as primary outcome), (iii) examine differences in body composition development up to 24 months of age (sum of skinfold thickness as primary outcome), and finally (iv) evaluate the long-term impact of the intervention formulas with assessments at 3, 4 and 5 years of age on growth and body composition (BMI-for-age z-score as main outcome parameter) as well as immunological, microbial and cognitive outcomes. A follow-up of the VENUS study was initiated to investigate this 4th objective. As indicated, for each of these objectives, a specific main outcome parameter was defined centred around growth and body composition development (see Table 1).

### 2.2. Setting

The VENUS (follow-up) study was conducted at the National University Hospital and the KK Women’s and Children’s Hospital in Singapore in compliance with the principles of the World Medical Association Declaration of Helsinki (59th WMA General Assembly, Seoul, Korea, October 2008), International Conference on Harmonisation (ICH) guidelines for Good Clinical Practice (GCP, September 1997), as well as Singapore’s regulatory requirements. This study and the associated follow-up were approved by the Domain Specific Review Board for the National University Hospital (approval no. 2011/01838) and SingHealth Institutional Review Board for KK Women’s and Children’s Hospital (approval no. 2011/635/E, follow-up: reference no: 2015/2418). The VENUS and VENUS follow-up study were registered at www.ClinicalTrials.gov (accessed on 19 August 2021)(identifier no. NCT01609634; NCT02594683).

### 2.3. Subjects and Study Design

Eligible subjects were healthy, term infants (gestational age ≥ 37 and ≤42 weeks), ≤28 days old of Chinese, Malay or Indian ethnicities or a mix thereof with a birth weight within the normal range for gestational age and sex (3rd to 90th percentile on the Fenton growth chart) [29] and a head circumference at birth within the normal range (3rd to 90th percentile on the Fenton chart). The infants recruited into the study had to live and intend to reside in Singapore for at least 2 years following recruitment. Initially, the study aimed to include only infants from Chinese ethnicity, but inclusion criteria were broadened following slow recruitment rates, after which infants of the 2 other main ethnic groups in Singapore, Malay and Indian, could be enrolled as well. 

Exclusion criteria were defined as infants with current or previous illnesses/conditions or interventions that could interfere with their growth, any known congenital diseases or malformations, which could interfere with study participation (e.g., gastrointestinal malformations, congenital immunodeficiency), those who require special diet other than a standard cow‘s milk-based IF, as well as infants who received any other IF for a duration longer than 10 days before inclusion/enrolment, and those with any history of or current participation in any other study involving investigational or marketed products. In addition, mothers with hepatitis B or human immunodeficiency virus (HIV) or with other significant medical conditions (including during pregnancy) that might interfere with the study were excluded. Finally, parents incapable of complying with the study protocol or investigator’s uncertainty about the willingness or ability of the parents to comply with the protocol requirements were not eligible to participate. Written informed consent was obtained from all parents of eligible subjects before enrolment. 

The study was designed as a randomised, double-blind, controlled intervention trial to investigate safety and tolerance of the concept IF in healthy term infants as well as its (longer-term) impact on their growth and body composition development. A cohort-like recruitment approach was adopted that allowed exclusive breastfeeding as well as any breastfeeding combined with formula feeding or full formula feeding during the first year of age. Overall, the design did not interfere with the choice in early milk feeding of infants; only when the parents made their decision to start formula feeding, their infants were randomized to receive a study product. In addition, after randomization, mothers were encouraged to continue breastfeeding. The formulas were coded by the sponsor using letter codes A, B, C, D, E and F with 2 letters for each type of milk; investigators and the infants’ parents were blinded to the study group allocation. Determination of which study product an infant received was based on a randomisation number that was generated using a computer random number generator with stratification for sex (male/female) and subject’s age at the time of randomisation (age ≤ 28 days, 29 days < age ≥ 17 weeks, or age > 17 weeks). An electronic case report form (eCRF) automatically provided the randomisation code when the respective eCRF information was completed. During the first 4 months of life, only breastfeeding or formula feeding (or a mix thereof) was allowed apart from the use of water, tea, rehydration solutions, drops or syrups (vitamins, minerals, medicines).

Based on randomisation age and breastfeeding duration, 3 different feeding groups were defined: a breastfeeding reference group (full breastfeeding at least until 17 weeks of age), a full formula feeding group (full formula feeding before or on 28 days of age) and a third “other-fed” group in which formula was introduced before 17 weeks of age (and not being fully formula-fed before 28 days of age).

### 2.4. Study Products

The intervention formulas were provided from randomisation during the first year of life as complete, standard cow’s milk-based formulas intended for infants until 6 months (IF) or from 6 to 12 months of age (follow-on formula; FOF). The IF study products were isocaloric (66 kcal/100 mL), contained similar levels of protein (1.3 g/100 mL with a whey:casein ratio of 60:40) and vegetable oil-based lipids (3.4 g/100 mL with a DHA:AA ratio of 1:2). Similarly, the FOF study products were isocaloric (66 kcal/100 mL), contained similar levels of protein (1.4 g/100 mL with a whey:casein ratio of 50:50) and vegetable oil-based lipids (3.0 g/100 mL with a DHA: AA ratio of 1:1). The key differences between the study IF/FOF products were the following: (1) the lipid droplet characteristics, and (2) a specific scGOS/lcFOS prebiotic mixture of short chain galacto-oligosaccharides and long-chain fructo-oligosaccharides (9:1; 0.8 g/100 mL). The concept group was provided with a standard IF/FOF with scGOC/lcFOS but containing lipid droplets having a volume-based mode diameter of 3–5 µm and an interface predominantly composed of milk phospholipids following an adapted production process [14]. In comparison, both control products comprised lipid droplets with a volume-based mode diameter of ~0.5 µm and had proteins as their main emulsifier. The Control group 1 was provided a standard IF/FOF containing the prebiotic mixture scGOS/lcFOS, whereas the Control group 2 was provided with the same standard IF/FOF without the prebiotic mixture.

### 2.5. Procedures and Assessments

Infants had an enrolment visit when they were ≤28 days of age, followed by visits at 1, 2, 3, 4, 6, 9, 12, 18 and 24 months of age, either at the clinic or at home (Table 2A), and yearly follow-up visits thereafter at 3, 4 and 5 years of age (Table 2B). After informed consent was obtained, participants’ demographic information, anthropometric data at birth and medical history were collected from medical records by study staff at the enrolment visit. At visit 1 (1 month of age), additional information on parental family and household characteristics, including medical, anthropometric and substance use data were gathered during an interview using a standardised form. Visit 2 (2 months of age) was only performed for infants randomized before or at 2 months of age. In the 10 days before visit 1 to 7 (52 weeks of age), parents filled-out a 7-day diary recording daily study formula intake and frequency of other feedings. In addition, they recorded gastro-intestinal complaints and stool characteristics. The severity of gastrointestinal symptoms, i.e., cramps, diaper rash, regurgitation and vomiting, was recorded once a day on a 4-point scale (1 = absent, 2 = mild, 3 = moderate, and 4 = severe). Stool consistency was scored for each stool passed, on a 4-point scale based on the use of pictures (1 = watery, 2 = soft, 3 = formed, 4 = hard) according to the “Amsterdam” stool form scale [30]. Instructions for introducing complementary foods were given according to regular (regional/national) guidelines for healthy subjects.

### 2.6. Growth and Fat Mass Development

Anthropometric measurements were collected at enrolment and each visit thereafter by trained study personnel following standardised procedures. Infants (naked) were weighed twice on calibrated electronic scales up to and including the age of 24 months and in light clothing without shoes for all time points thereafter. Supine length of infants was measured twice using a standard measuring board (Seca 210 Mobile Measuring mat), while standing height measurements using standardised stadiometers were allowed from 18 months onwards. To measure head circumference, a non-stretchable measuring tape was used in duplicate. If the anthropometric measures deviated substantially (>100 g for weight and >5 mm for length and head circumference), a third measurement was obtained. Mid-upper arm circumference was measured using a non-stretchable measuring tape in duplicate. Biceps, triceps, subscapular and suprailiac skin fold thicknesses of the infants were measured from 3 months using a standard protocol and calibrated skinfold caliper. At 24 and 60 months of age, ultrasound measurements to assess visceral and subcutaneous abdominal body fat mass (preperitoneal distance, preperitoneal area, subcutaneous transversal distance and subcutaneous area) were taken based on the approach validated in the Generation R study [31,32,33,34].

### 2.7. Safety and Tolerance

After enrolment until 24 months of age, parents were asked to record subjects’ frequency and severity of adverse events, medication use or visit(s) to a health care professional, which was discussed during the clinic visits. During the phone calls, parents were asked if there were any occurrences or changes in their infants’ experiences of illness or medical symptoms, medication use and visit(s) to a health care professional since the last assessment. The information on medical events (including fever or elevated body temperature, subsequent medication and treatment), as well as daily recording of intake of human milk, study product, complementary food (if any), stool frequency and consistency, as well as the occurrence and severity of gastrointestinal symptoms recorded in the diary 7 days prior to a study visit was used to review the subject’s tolerance to the study product and evaluation of the severity of occurred (serious) adverse events (AEs) during each visit. 

### 2.8. Exploratory Outcomes during Follow-Up at 3, 4 and 5 Years of Age

During the follow-up period, we aimed, in addition to growth and body composition, to obtain insights into eating style or dietary pattern, using the Children Eating Behaviour Questionnaire (CEBQ) and a Food Frequency Questionnaire (FFQ). The CEBQ is a parent-reported questionnaire consisting of 35 items, each rated on a five-point Likert scale. It is made up of 8 scales that provide a comprehensive overview of eating styles [35,36]. The FFQ is typically designed to assess the habitual intake of individual food items or specific food groups over a period of time (typically 6 or 12 months) in a population of interest [37,38]. The FFQ used in this FU study asked for the frequency of intake, categorized from ‘never’ to ‘>6 times per day’ of a total of 110 food items over the past 3 months, providing descriptive qualitative information about food-consumption patterns, and was specifically designed for Singaporean toddlers. The ethnic-specific food lists used in this FFQ were based on those developed in a study with 30 mothers of toddlers (10 from each of the major ethnic groups in Singapore) who completed 3-day food records and participated in focus groups [39]. 

Finally, we aimed to obtain insights in brain development using a modified NIH Toolbox Early Childhood Cognition Battery for assessment of cognitive development [40,41]. The selected battery, recommended for ages 3–6, included the Dimension Change Card Sort (DCCS), Flanker, and Picture Sequence Memory measures, which assessed attention, memory and executive function. DCCS is a measure of cognitive flexibility using target pictures that vary along two dimensions (e.g., shape and colour). Test scoring is based on a combination of accuracy and reaction time and takes approximately 4 min to complete. The Flanker task measures both attention and inhibitory control. The test requires the participant to focus on a given stimulus while inhibiting attention to other stimuli. Test scoring is based on a combination of accuracy and reaction time and takes approximately 3 min to complete. Finally, the Picture Sequence Memory Test is a measure developed for the assessment of episodic memory and involves recalling an increasingly lengthy series of illustrated objects and activities presented in a particular order on the computer screen and takes approximately 7 min to complete.

### 2.9. Biological Samples

Biological samples (stool, saliva and buccal smear) were collected at the time of randomisation and at 1, 3, 6 and 12 months as well as at 3, 4 and 5 years of age to explore the composition and metabolic activity of the intestinal microbiota and immunological and epigenetic biomarkers associated with the risk of developing an allergy. On a voluntary basis (a subset of subjects) a blood sample was collected at the age of 12 months as well as at 5 years of age. At 12 months of age, the blood sample was to be analysed for immunological parameters, blood levels of fat-soluble vitamins, liver function and kidney function test (alanine aminotransferase (ALAT), aspartate aminotransferase (ASAT), alkaline phosphatase (ALP), gamma-glutamyl transpeptidase (GGT), albumin and creatinine levels). At 2 visits, at 24 and 60 months of age, a skin prick test was performed. Parents were asked to contact the study site if development of eczema/atopic dermatitis was suspected at any time during the study. Diagnosis of atopic dermatitis cases was based on criteria by Hanifin and Rajka [42]. In case a subsequent episode of eczema/atopic dermatitis occurred after positive diagnosis of atopic dermatitis was made, subsequent diagnostic assessment was not required.

### 2.10. Statistics

As indicated previously, instead of defining a single overall primary outcome parameter for the study, it was decided to pre-define primary outcome parameters specifically related to each of the 4 objectives of the study, all centred around growth and body composition development (see Table 2). The required sample size was calculated for the first 2 objectives within the intervention period (0–12 months of age). 

The first objective was to evaluate the safety of the concept formula. To this end, we aimed to demonstrate equivalence of daily weight gain (g/day) between baseline and 17 weeks of age in infants consuming the Concept versus Control 1 formula, both containing the specific prebiotic mixture. It is of importance to note that since the objective is to evaluate the safety of the formulas, this analysis will, in line with guidelines, only include those infants that have been fully formula-fed at 28 days of age (latest). Equivalence was to be demonstrated when the 2-sided 90% CI of the difference in means of daily weight gain lay within the pre-defined margin ± 3 g/day [43]. The required sample size for 2 1-sided statistical tests based on an SD of 6.0 g/day (16), α = 0.05, a power = 0.80, and assuming no daily weight gain differences between the 2 intervention groups, was 70 infants per intervention group. Assuming a drop-out rate of 15%, a total of 249 infants (83 per group) was needed. Based on unpublished data from an ongoing cohort study in Singapore at that time, we estimated that 27% of the infants would be fully formula-fed by 4 weeks of age. Therefore, a total sample size of 922 subjects was estimated to include 249 subjects in the “fully formula-fed group”. Consequently, the remaining subjects would contribute either to the “fully breastfed group” or “other-fed group”, depending on the timing of randomisation. 

The second objective was to evaluate differences in infant growth outcomes between infants fed Concept vs Control 1 formula, both containing the specific prebiotic mixture, during the first year of life, with weight gain between birth and 12 months of age as a primary outcome parameter. It was hypothesized that infants consuming the Concept formula would have a growth pattern, i.e., weight gain, closer to that observed in breastfed infants. The required sample size using a two-sided t-test at a 5% level of significance with 80% power was 201 subjects (67 subjects/arm, 3 arms). This was based on detection of an assumed mean difference of 472.4 g in weight gain (as observed between formula-fed and breastfed infants, with SDs for the two groups of 998.3 and 926.7 g, respectively) from birth up to 1 year of age, between infants consuming the Concept versus Control 1 formula (randomised before 4 months of age). The assumption for weight gain difference (effect size) as well as SDs were derived from historical data from a previous clinical trial, including both formula and breastfed infants [44]. Finally, with an assumption of 15% drop-out rate, a total estimate of 237 subjects (79 subjects/arm) were required for this part of the study.

Apart from the growth equivalence analysis, which comprises fully formula-fed (<28 days of age) infant population only, the remainder of the primary outcome analyses (objectives ii–iv) will be performed using data of all subjects randomised to infant formula before 17 weeks of age. Infants who were either randomised after 17 weeks of age or not randomised at all were considered to serve as a breastfed reference group. 

### 2.11. Interim Analysis

An interim analysis was conducted during the study to evaluate the safety/tolerance of the study products and address any issue(s) that could potentially impact the continuation of the study. An independent Data Monitoring Committee (DMC) was established to review safety data and provide recommendations on the results of the interim analysis. This interim evaluation was performed 12 months after recruitment had started, and the results were presented to the DMC in March 2014. In addition, assessment of 17-week equivalence study (tolerance, safety and growth collected up to 27 February 2015) on infants in all groups were presented in September 2015. Based on the outcomes of these assessments, the DMC recommended no change in the protocol and the VENUS study continued as planned. 

## 3. Results

The study was initially designed to include a total of 922 subjects based on the power calculation for the equivalence study (objective i) and the assumed rate (27%) of full formula feeding in Singapore. After the start of the study, it became evident that feeding practises in the recruited population were not reflecting the assumed dichotomy in either full formula or full breastfeeding, but often were more longer-term mixed feeding practices. Consequently, it would have required many more recruited infants given the cohort-based recruitment strategy to fulfil the original aim of including 249 fully formula-fed infants (before 28 days of age) for the equivalence analysis. This, together with unforeseen ingredient sourcing issues for the study products, led to a premature stop in recruitment, still ensuring study product supply for subjects who had already been enrolled in the study. For the sake of clarity, it should be emphasized that no safety concerns were related to this decision to stop the inclusion of subjects, but that the decision was based on unrealistic feasibility and acknowledged by the DMC in March 2014. As a consequence, the equivalence analysis linked to the first objective of the study will be underpowered. Although the total number of eligible and enrolled subjects (see details below) will likely be sufficient for efficacy analysis, the effect of the large variation in (mixed) feeding practices on the (growth) outcomes in the intervention arms remains to be elucidated. The VENUS study and its follow-up have completed recruitment and assessment with the last participant completing the 24-month visit in August 2016 and the last 5-year assessment in the follow-up period completed in August 2019.

**Figure 1:**
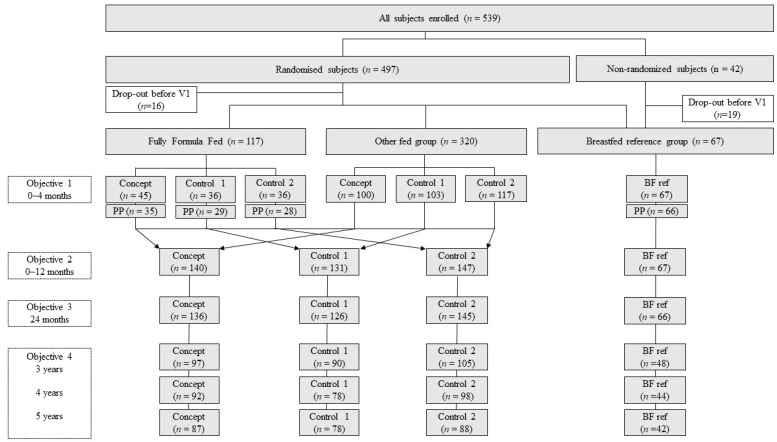
Flow chart of the progression of infants during the study. Fully formula-fed, infants that were fully formula-fed latest at 28 days of age; other fed group, infants randomised before 4 months of age; BF ref, infants that were fully breastfed at least until 4 months of age; concept, a standard IF/FOF with the prebiotic mixture scGOC/lcFOS and containing large lipid droplets coated with milk phospholipids; Control 1, a standard IF/FOF containing the prebiotic mixture scGOS/lcFOS; Control 2, a standard IF/FOF without the prebiotic mixture. Objective 1—growth equivalence analysis (daily weight gain 0–4 months as primary outcome); Objective 2—growth efficacy analysis between birth and 12 months (weight gain 0–12 months as primary outcome); Objective 3—body composition development up to 24 months of age (sum of skinfolds as primary outcome); Objective 4—evaluate long-term impact on growth and body composition (BMI-for-age z-score as main outcome parameter). PP, per protocol population.

A total of 590 healthy term infants were screened between June 2012 and July 2014. Of these, 539 infants were eligible and enrolled in the study (Figure 1). In total, 90% of the eligible infants completed the 1-year assessment [*n* = 485]. At 3 years, 63% of these infants [*n* = 340] started with the yearly follow-up, decreasing to 58% [*n* = 312] and 55% [*n* = 295] retainment at, respectively, 4 and 5 years of the original study population. Infants recruited in the VENUS study included 337 Chinese (62.5%), 161 Malay (29.8%), 24 Indian (4.5%) and 17 mixed ethnicities (3.2%)] (Table 3). 

Lost to follow-up in the VENUS study and during its follow-up period was comparable between study product arms. The mean gestational age and birth weight of the enrolled infants were 272 days and 3154 g, respectively. Thirty-five subjects dropped out from the trial before the age of 28 days (visit 1) due to consent withdrawal, loss to follow-up, serious AE (a case of severe regurgitation assessed as probably related to the study product by the investigator), as well as other reasons that included changes in residencies. No apparent differences in the infant or maternal demographics were observed between the drop-outs and the remaining infants in the study (data not shown).

Only 117 infants of the 497 infants consuming formula were fully formula-fed at 28 days of age, and as such, eligible for the equivalence analysis related to the first study objective. In total, 336 infants were randomised to formula before 4 months of age but were not fully formula-fed at 28 days of age, ending up in the other-fed group. Lastly, the Breastfed Reference group, defined as exclusively breastfed up to 17 weeks of age, was comprised of 67 infants, of which 23 were not randomised at all during the first 12 months of age (12.4% and 4.3%, respectively, of the total enrolled infant population). 

## 4. Discussion

The WHO recommends exclusive breastfeeding for 6 months as the optimal way of feeding infants, where they receive only breast milk with no other liquids/water/solids, with the exception of oral rehydration solution, drops/syrups of vitamins, minerals or medicines [1]. Despite these guidelines, rates of exclusive breastfeeding for the first 6 months remain low. For example, the rates of 6-month exclusive breastfeeding in the United States, Australia and Taiwan are 18.8%, 17.6% and 24.3%, respectively [45,46,47]. Recently the Growing Up in Singapore Toward healthy Outcomes (GUSTO) cohort reported a prevalence of full breastfeeding at 6 months of 11%, 2% and 5% in Chinese, Malay and Indian mothers, respectively [48]. Globally, maternal smoking and education level, but more importantly, the need to return to work [49,50,51,52], are some of the main obstacles accounting for the low rates of exclusive breastfeeding observed.

We implemented a cohort-like recruitment in the VENUS study, based on available data on breastfeeding practise from the National University Hospital and KK Women’s and Children’s Hospital in Singapore. Our approach, allowing randomisation of study subjects according to the parents’ autonomous decision to start their infants on formula or not at any time throughout the course of the study, is quite novel. Since infants are only randomised into one of the study arms at the moment, their caregivers decided to use IF/FOF, this study design represents a pragmatic approach that will allow the study to account for different exposure, start and duration of formula feeding during the first year of life, as compared to conventional study designs. Given the successful implementation of Baby-friendly Hospital Initiative (BFHI) recommended by WHO and the United Nations Children’s Fund (UNICEF) around the world [4], the rate of (exclusive) breastfeeding has increased, and duration of any type of breastfeeding has become much higher [51], also in Singapore [52,53]. Besides the fact that conducting a clinical study on exclusive formula feeding (and including an exclusive breastfeeding reference group) represents ethical and logistic challenges, the current approach offers a unique opportunity to explore the impact of feeding practice that combines breast and formula feeding, which may be used to examine the impact of feeding practice, patterns and characteristics on growth and to develop educational support.

There are, however, several potential limitations to the cohort-like design of the VENUS study. First, upon enrolment, the subjects’ parents were made aware that there was an IF readily available for them to use as soon as they decide to switch to formula feeding. This may have influenced their decision to select the source of nutrition for their infants and differ from a conventional cohort setting. In our study, indeed, most subjects belong to the “other-fed group”, compared to much smaller numbers recruited in both the “fully formula-fed group”, as well as in the “breastfed reference group” (defined as exclusively breastfed for at least 17 weeks). Interestingly, the proportion of subjects who received any breastfeeding was higher in this study than in the GUSTO cohort [48], suggesting that the design of the VENUS study did not negatively influence infant feeding practices in Singapore. This could partially be due to the effective implementation of the BFHI in Singapore although the impact of BFHI implementation on the start and duration of breastfeeding remains to be investigated.

In addition, as recruitment was stopped early to ensure a sufficient supply of study products for all already enrolled subjects in the study, the number of study subjects was reduced significantly compared to our initial sample size calculation for growth equivalence. In addition, the subsequent recruitment of subjects of non-Chinese ancestry resulted in the heterogeneity of the study population, although this was limited to the two other main ethnicities, Malay and Indian. Given known differences in growth trajectories between Asian populations [54], the original design of the study aimed to recruit a more homogenous population of Chinese descent only. Both the reduction in the total number of study subjects and inclusion of study subjects from different ethnicities may result in loss of study power and increase subject heterogeneity.

## 5. Conclusions

In conclusion, the VENUS study was designed to explore the potential contribution of concept formula with large, milk phospholipid coated lipid droplets comprising a vegetable oil-based fat fraction on growth and early body composition development in healthy infants. The design of the study and recruitment strategy represents a pragmatic and breastfeeding supportive approach that is truly different from the standard clinical intervention trial design. Apart from the evaluation of the safety and efficacy of the concept formula, the insights from this study may contribute to further discussions on the development of new approaches in clinical study design to test new infant formula innovations in the future while respecting breastfeeding practices.

## Figures and Tables

**Table 1:** Growth and body composition outcome measures from VENUS and VENUS follow-up study.

Outcomes	Objectives 1–3			Objective 4		
	Equivalence and Safety (Start of Randomization—17 Weeks)	Efficacy (Birth—12 Months)	Follow-Up (Birth—24 Months)	Follow-Up (3 Years)	Follow-Up (4 Years)	Follow-Up (5 Years)
Weight gain/day	#	-	-	-	-	-
Total weight gain	-	#	-	-	-	-
Body Weight	-	-	X	X	X	X
Recumbent length, head circumference, mid-upper arm circumference and skin folds **	X	X	X	X	X	X
Sum of skin fold thickness ^§^	X	X	#	X	X	X
Visceral and subcutaneous abdominal body fat mass ^@^	-	-	X	-	-	X
Gastrointestinal tolerance	X	X	-	-	-	-
AE and SAE	X	X	X	-	-	-
Weight-for-age z-scores	X	X	X	X	X	X
Weight-for-length z-scores	X	X	X	X	X	X
Length-for-age z-scores	X	X	X	X	X	X
BMI-for-age z-scores	X	X	X	#	#	#
Head circumference-for-age z-scores	X	X	X	X	X	X
Mid-upper arm circumference-for-age z-scores	X	X	X	X	X	X
Skinfold thickness-for-age z-scores (subscapular and triceps)	X	X	X	X	X	X

AE: adverse event; SAE: serious adverse event. # Indicates primary outcome measure; ** Skin folds measurements include subscapular, triceps, biceps and suprailiac.; ^§^ Sum of skin fold thickness includes triceps, biceps, suprailiac and subscapular. ^@^ Measurements performed by ultrasound and they include preperitoneal distance and area, as well as subcutaneous transversal distance and area.

**Table 2:** (**A**) Visit schedules for VENUS study up to 24 months of age. (**B**) Visit schedule for VENUS follow-up study at 3, 4 and 5 years of age.

**(A)**													
**Procedures and Assessments**	**Start of Study**		**Intervention**							**Follow-Up**			
	**Screening** **Up to 28 Days of Age**	**Randomization** **When Parent(s) Decide to Start Formula + 3 Days**	**Visit 1** **(Home)** **4-Week** **± 2 Days**	Visit 2 * **(Home)** **8-Week** **± 5 Days**	**Visit 3** **(Home)** **13-Week** **± 5 Days**	**Visit 4** **(Home)** **17-Week** **± 5 Days**	**Visit 5** **(Home)** **26-Week** **± 10 Days**	**Visit 6** **(Home)** **39-Week** **± 10 Days**	**Visit 7** **(Clinic)** **52-Week** **± 10 Days**	**Phone Calls** **65-Week** **± 14 Days**	**Visit 8** **(Home)** **78-Week** **± 14 Days**	**Phone Calls** **91-Week** **± 14 Days**	**Visit 9** **(Clinic)** **104-Week** **± 14 Days**
Informed consent	x												
Screening/eligibility check	x	x											
Demographic data	x												
Parental characteristics			x										
Family characteristics			x		x		x		x				x
Feeding practice		x	x	x	x	x	x	x	x				
Medical history and pre-existing condition	x												
Birth weight, length and head circumference	x												
Weight, length, head and mid-upper arm circumferences		x	x	x	x	x	x	x	x		x		x
Skin-fold thickness					x	x	x	x	x		x		x
Stool, saliva and buccal swap		x	x		x		x		x				
Dispense diary on medication	x		x	x	x	x	x	x	x		x		
Dispense diary on intakes and stool characteristics	x		x	x	x	x	x	x					
Diary collection			x	x	x	x	x	x	x		x		x
Product acceptance questionnaire							x		x				
Ultrasound measurement													x
Skin prick test													x
Blood samples ^§^									x				
AE and SAE	Recorded after the informed consent was obtained and throughout the study												
Concomitant medication, including nutritional supplements	Recorded throughout the study												
Allergic manifestation (including atopic dermatitis)	Recorded throughout the study												
**(B)**													
**Procedures and Assessments**	**Enrolment Visit** **(V10, V11 or V12)**		**Visit 10 (Clinic)** **36 Months ± 2 Months**		**Phone Call 3** **42 Months** **± 1 Month**			**Visit 11 (Clinic)** **48 Months ± 2 Months**		**Phone Call 4** **54 Months** **± 1 Month**		**Visit 12 (Clinic)** **60 Months ± 2 Months**	
Informed consent	X *												
Screening/Eligibility check	X												
Subject and Family characteristics	X												
Medical history, pre-existing condition (ongoing cases only)	X												
Anthropometric measurements (weight, length, head, and mid-upper arm circumferences, skin-fold-thickness)			X					X				X	
NIH Toolbox Early Childhood Cognition Battery			X					X				X	
Child Eating Behaviour Questionnaire			X					X				X	
Food Frequency Questionnaire	X											X	
Ultrasound Body Composition			X **^,#^									X	
Skin Prick Test			X **^,#^									X	
Allergy Questionnaire			X					X				X	
Infection Questionnaire			X					X				X	
Collection of saliva samples			X					X				X	
Collection of stool samples			X (or within 5 days after V10)					X (or within 5 days after V11)				X (or within 5 days after V12)	
Collection of blood samples (optional)												X	
(S) AEs	Recorded throughout the study period												
Incidence of infection	Recorded throughout the study period												
Incidence of allergic manifestation	Recorded throughout the study period												
Usage of relevant medications	Recorded throughout the study period												

(**A**) AE: adverse event; SAE: serious adverse event; * Time point only for subjects who were randomized before or at 2 months of age; ^§^ Samples were collected on voluntary basis only. (**B**) * Informed consent can be obtained during screening period (V9) or at Enrolment Visit; ** For those subjects who missed the assessments when they are 2 years old; ^#^ Both the ultrasound measurement and skin prick test will not be performed if the visits are conducted at home.

**Table 3:** Demographic characteristics of study participants.

			Randomised			Non-Randomised
Category	Statistic	Enrolled Subjects *n* = 539	Fully Formula-Fed *n* = 117	Other-Fed Group *n* = 320	Breastfed Reference Group *n* = 44	Breastfed Reference Group *n* = 23
**Infant**						
Ethnicity						
Chinese	*n* (%)	337 (62.5%)	52 (44.4%)	212 (66.3%)	36 (81.8%)	20 (87.0%)
Malay	*n* (%)	161 (29.9%)	57 (48.7%)	81 (25.3%)	5 (11.4%)	3 (13.0%)
Indian	*n* (%)	24 (4.5%)	1 (0.9%)	18 (5.6%)	3 (6.8%)	0 (0.0%)
Other	*n* (%)	17 (3.2%)	7 (6.0%)	9 (2.8%)	0 (0.0%)	0 (0.0%)
Sex						
Male	*n* (%)	279 (51.8%)	68 (58.1%)	162 (50.6%)	21 (47.7%)	9 (39.1%)
Female	*n* (%)	260 (48.2%)	49 (41.9%)	158 (49.4%)	23 (52.3%)	14 (60.9%)
Age at start of study product (days)						
	Mean (SD)	30.21 (62.11)	5.84 (4.68)	15.36 (21.51)	212.00 (60.76)	--
Gestational age (days)						
	Mean (SD)	272.92 (7.05)	271.96 (7.09)	273.13 (6.91)	271.66 (6.71)	275.13 (9.20)
Birth weight (grams)						
	Mean (SD)	3154.64 (352.26)	3100.98 (342.89)	3173.64 (358.29)	3125.36 (279.58)	3163.52 (360.63)
Mode of delivery						
Vaginal	*n* (%)	370 (68.6%)	84 (71.8%)	214 (66.9%)	34 (77.3%)	17 (73.9%)
Caesarean	*n* (%)	137 (25.4%)	28 (23.9%)	85 (26.6%)	6 (13.6%)	5 (21.7%)
Instrumental	*n* (%)	32 (5.9%)	5 (4.3%)	21 (6.6%)	4 (9.1%)	1 (4.3%)
**Mother**						
Age (years)	Mean (SD)	30.22 (5.02)	28.89 (5.88)	30.84 (4.76)	30.66 (3.74)	30.17 (3.89)
Education level						
Primary School	*n* (%)	16 (3.2%)	10 (8.6%)	5 (1.6%)	0 (0.0%)	1 (4.3%)
High school/trade school or equivalent	*n* (%)	307 (61.0%)	95 (81.9%)	195 (61.1%)	15 (34.1%)	2 (8.7%)
University (above)	*n* (%)	179 (35.6%)	11 (9.5%)	119 (37.3%)	29 (65.9%)	20 (87.0%)
Unknown	*n* (%)	1 (0.2%)	0 (0.0%)	0 (0.0%)	0 (0.0%)	0 (0.0%)
Number of previous pregnancies						
0	*n* (%)	138 (27.5%)	23 (19.8%)	94 (29.5%)	9 (20.5%)	12 (52.2%)
≥1	*n* (%)	364 (72.5%)	93 (80.2%)	225 (70.5%)	35 (79.5%)	11 (47.8%)

## Data Availability

Not applicable.
